# Predictive Value of Second-Trimester Maternal Lipid Profiling in Early-Onset Pre-eclampsia: A Prospective Cohort Study and Nomogram

**DOI:** 10.3389/fmed.2021.688312

**Published:** 2021-12-01

**Authors:** Juan Li, Juefei Lu, Mengni Wang, Wen Hu, Neng Jin, Xingmiao Li, Baihui Zhao, Qiong Luo

**Affiliations:** Department of Obstetrics, Women's Hospital, Zhejiang University School of Medicine, Hangzhou, China

**Keywords:** lipid profile, prediction, early-onset pre-eclampsia, nomogram, bootstrap

## Abstract

**Purpose:** Maternal lipid profile in second trimester has rarely been investigated in the risk assessment for pre-eclampsia (PE). Since early-onset PE often companied by much worse clinical outcomes, thus, we aimed to evaluate the predictive value of second-trimester maternal lipid profiling for early-onset PE.

**Methods:** A prospective cohort study was conducted to measure the second-trimester maternal lipid profile of pregnant women from January to December 2019. The pairwise association between maternal lipid profile and PE onset or pregnancy termination time was quantified. Multiple logistic regression was preformed to define risk factors for early-onset PE, and a nomogram for early-onset PE was developed. The net benefit of our model was evaluated by calibration curve and decision curve analyses.

**Results:** We enrolled 5,908 pregnant women and they were divided into healthy (*n* = 5,789), late-onset PE (*n* = 64), and early-onset PE (*n* = 55) groups. Total cholesterol (TC), triglycerides (TG), and low-density lipoprotein cholesterol (LDL-c) were elevated in patients with PE, while high-density lipoprotein cholesterol (HDL-c) was decreased in patients with PE. TC, TG, and LDL-c were negatively correlated with PE onset time or gestational week at delivery. Receiver operating characteristic curves (ROC) defined the cutoff values of TG and HDL-c, and the final regression model included five statistically significant risk predictors for early-onset PE (maternal age of ≥35 years, multipara, pre-pregnancy body mass index (BMI) ≥25 kg/m^2^, second trimester TG ≥ 2.59 mmol/L and second trimester HDL-c ≤ 2.03 mmol/L. The nomogram had an excellent diagnostic performance (area under the curve = 0.912, sensitivity = 92.7%, and specificity = 76%) and was further validated with good calibration and positive net benefits in a decision curve analysis.

**Conclusions:** An abnormally increased TG concentration and a decreased HDL-c concentration might serve as predictors of early-onset PE. Whether blood lipid-lowering measures can improve severe PE prognosis require further clarification.

## Introduction

Pre-eclampsia (PE) is defined as new-onset hypertension (>140/90 mmHg) occurring after the 20th week of gestation accompanied by one or more of new-onset proteinuria, maternal organ dysfunction, or uteroplacental dysfunction (1). The international incidence of PE is 3–5% of all pregnancies (2), causing maternal and neonatal morbidity and mortality globally.

PE is a complex disorder, and its pathogenesis is still not fully understood. At present, it is believed that PE begins during trophoblast development and invasion. The process might result in neovascularization and placental dysfunction, which consequently cause release of humoral substances into the maternal circulation, leading to clinical manifestation (3), while some other scholars believed that abnormal pre-pregnancy maternal cardiovascular function may lead to trophoblast malperfusion and may be the origin of PE (4) PE can be roughly divided into early-onset and late-onset subtypes, each with its etiopathogenic background (5). Early-onset PE is defined as disease developing before 34 weeks of gestation and is usually associated with an increased risk of adverse pregnancy outcomes, severe pre-eclampsia with maternal end-organ damage. Any treatments that prolong pregnancy would enable the fetus to mature further and reduce the maternal organ damage are thus considered beneficial.

Lipids are significantly elevated in pregnancy with a noticeable increase during the late trimester (6). The most prominent change is a 2.7-fold increase in triglycerides in the third trimester (7–9). This occurs mainly during the third trimester of pregnancy to ensure supply of nutrients and essential fatty acids in adequate quantities to the placental–fetal unit (10). Previous research shows that elevated maternal blood lipids are positively correlated with the risk of coronary heart disease (CHD); while high-density lipoprotein cholesterol (HDL-c) is recognized as an independent protective factor against CHD (11).

Women with PE have biologically exaggerated lipid secretion. A recent meta-analysis of 74 studies found that PE is associated with elevated total cholesterol (TC) and non-HDL-c in the third trimester (12); however, data are still conflicting and may be limited by the very small sample sizes of women with PE. The role of serum lipids in PE onset is uncertain. Changes in lipid metabolism may be an expression of placental insufficiency and diminished fetal growth. Some studies suggest that dyslipidemia with increased serum triglycerides (TGs) in early pregnancy before 20 weeks of gestation and elevated oxidized low-density lipoproteins (LDLs) are associated with an increased risk of PE (13–16). Very few studies have focused on the relationship between the lipid levels in the second trimester and pregnancy comorbidities, but in fact, as a hierarchical diagnosis and treatment plan, pregnant women often enter a large hospital for management from the second trimester. There are currently no worldwide standard criteria for lipid levels during pregnancy due to population and territory heterogeneity, Some authors have tried to establish reference ranges for maternal lipids, and they found that gestational hyperlipidemia may be associated with metabolic morbidities (6, 17, 18).

To date, hypolipidemic drugs have been proven as safe to use during pregnancy (19); however, they are not popularized in the clinic. This may be due to lack of unified criteria for the evaluation of their effectiveness. Whether or not there is a correlation between second trimester lipid profile and time of PE onset or improvement in severe PE prognosis still needs to be elucidated. In this study, we aimed to explore the predictive value of abnormal second-trimester maternal lipid profiling for early-onset PE. We constructed a visualized, intuitive, and appreciable nomogram that can be used to facilitate practitioners' medical performance. This nomogram may help to develop strategies for prenatal severe PE management.

## Methods

### Study Population

This prospective single-center pilot study was conducted over a period of 1 year at Women's Hospital, Zhejiang University School of Medicine, from January 2019 to December 2019. Pregnant women were enrolled from 24 to 28 weeks of gestation, as defined by first trimester ultrasound. The study enrolled women aged 20–45 years. All patients were followed up until delivery and observed for PE development. The study was approved by the ethics committee of Women's Hospital, Zhejiang University School of Medicine (IRB-2021003-R).

**Figure 1:**
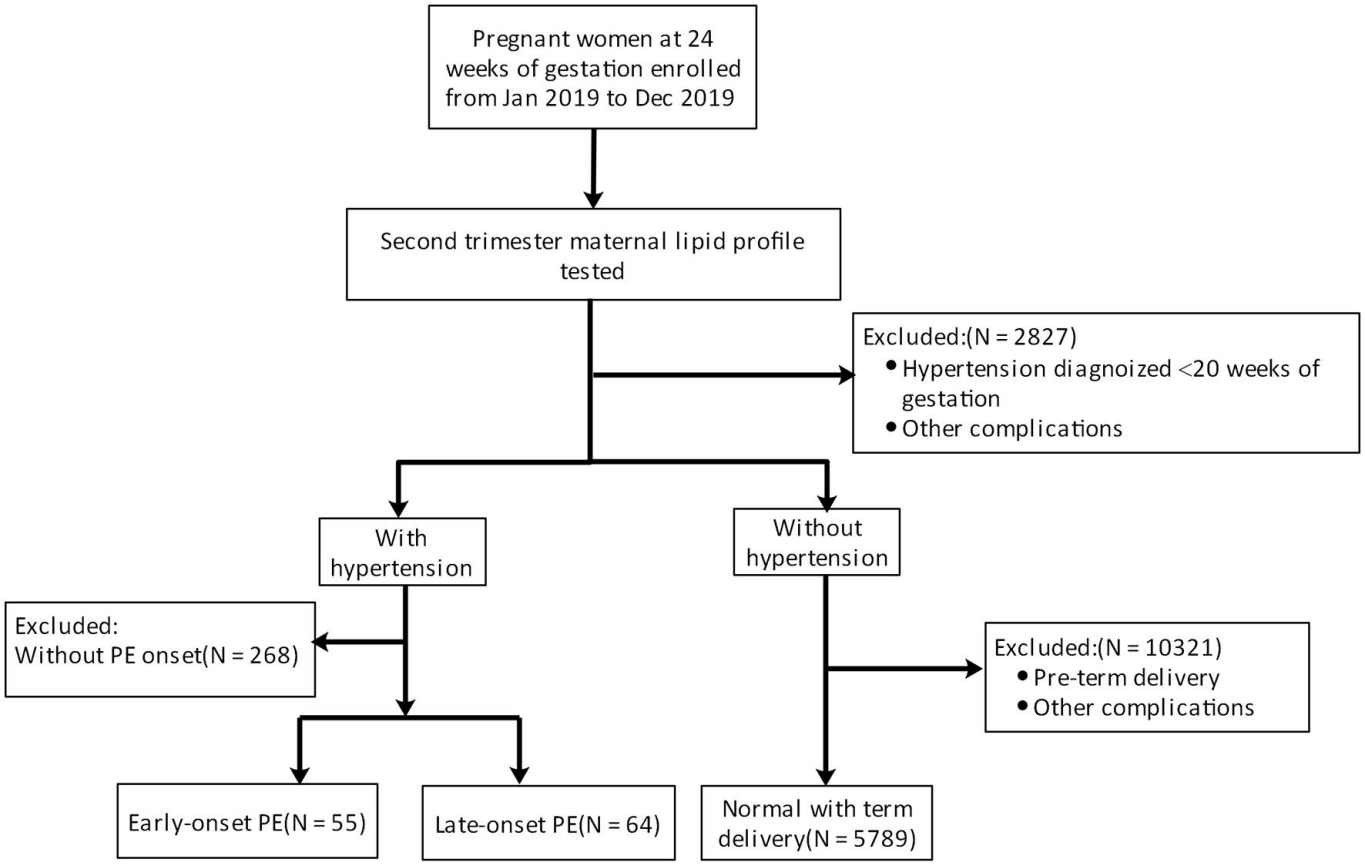
Pregnant women were first enrolled (excluding those with hypertension or other comorbidities) at 24 weeks of gestation. Then, the lipid profile was evaluated. Pregnant women were classified into a healthy group, an early-onset PE group, and a late-onset PE group by observing PE development.

PE is defined as new-onset hypertension (>140/90 mmHg) and proteinuria (>300 mg/d) after the 20th week of gestation or absence of proteinuria but fulfillment of other criteria for PE according to the American College of Obstetricians and Gynecologists' 2019 Practice Bulletin (Gestational Hypertension and Pre-eclampsia). In the present study, patients with PE were divided into early-onset PE (delivered before 34 weeks of gestation) and late-onset PE (delivered after 34 weeks of gestation) groups. Women with uncomplicated pregnancies and full-term delivery constituted the healthy group. The exclusion criteria were as follows: (1) multiple pregnancy; (2) diabetes mellitus or gestational diabetes; (3) chronic kidney disease; (4) systemic inflammatory disorders; (5) premature delivery for other reasons; (6) hypertension diagnosed before 20 weeks of gestation; (7) any medical disease that may alter lipid profile; (8) use of medications to regulate glucose or cholesterol (Figure 1). Fetal growth restriction (FGR) was defined as a birth weight <10th centile with elevated umbilical artery Doppler systolic/diastolic ratio or a resistance index >95th centile for gestation (20).

All patients underwent fasting lipid profiling in the second trimester (24–28 weeks). TC, TG, and HDL-c were measured using the Beckman Coulter AU5800 automatic biochemical analyzer according to standard laboratory methods, while LDL cholesterol (LDL-c) was calculated using the Friedewald formula (21). The fasting time for the sample collection ranged from 6 to 12 h. Therapy details and final clinical outcomes obtained thereafter were recorded and analyzed.

### Statistical Analysis

Data are summarized using routine descriptive studies for numerical variables, while categorical variables are summarized as counts and percentages. The Shapiro–Wilk test was used to measure the normality of distribution of the continuous variables. The Kruskal–Wallis test was performed by comparing individual lipid profile parameters among different groups and other ordinal variables. When the *P*-value from the Kruskal–Wallis test was statistically significant, a multiple comparison test was performed to determine which groups differed from which other groups. The Chi-squared test was used to evaluate nominal variables, and Spearman's rank correlation was used to quantify the pairwise association between each of TC, TG, HDL-c, LDL-c, and onset of PE or pregnancy termination time of PE. Linear discriminant analysis was performed to introduce multiple logistic regression analysis to define risk factors for early-onset PE, and adjusted odds ratios (ORs) and 95% confidence intervals (CIs) were calculated. Collinearity analysis was performed on all independent variables to eliminate the variable with a variance inflation factor >10. *P* < 0.05 were considered statistically significant.

### Nomogram Construction and Validation

A nomogram of early-onset PE was developed using forward stepwise multiple logistic regression. The prediction model that incorporated the identified predictors was completed and presented as the nomogram. The each predictor point is first determined by drawing a vertical line to the points axis and then add the points of each predictor; finally, draw a line from the total point axis to determine the estimated early-onset PE probabilities at the lower line of the nomogram.

Receiver operating characteristic (ROC) curve analysis was used to evaluate the discriminatory ability of the model. Then, the accuracy of the model was further verified by bootstrap validation using computer resampling for 1,000 repetitions of simple random sampling with replacement. The calibration curve was employed to detect the concentricity between the model probability curve and the ideal curve. A decision curve analysis was also performed to evaluate the net benefit of the model. All tests were two-sided with an alpha level of 0.05.

Statistical analyses were performed using SPSS 26.0 (IBM Corp., Armonk, NY) and R (http://www.Rproject.org).

## Results

### Demographic Characteristics of the Study Population

Pregnant women were divided into healthy (*n* = 5,789), late-onset PE (*n* = 64), and early-onset PE (*n* = 55) groups. Maternal demographic data for all maternal samples are presented in Table 1.

**Table 1:** Demographic and clinical characteristics of the three study groups.

	**Healthy (*N* = 5,789)**	**Late-Onset PE (*N* = 64)**	**Early-Onset PE (*N* = 55)**
Maternal age (years)	28.8 ± 3.2	31.4 ± 4.6*	32.6 ± 4.2*
**Gravidity**			
≥2	37.5% (*N* = 2,168)	47.0% (*N* = 31)	79.9% (*N* = 39)^*^^#^
≤ 1	62.5% (*N* = 3,621)	53.0% (*N* = 35)	29.1% (*N* = 16)^*^^#^
**Parity**			
≥1	10.6% (*N* = 614)	24.2% (*N* = 16)^*^	56.4% (*N* = 31)^*^^#^
0	89.4% (*N* = 5,175)	75.8% (*N*=50)^*^	43.6% (*N* = 24)^*^^#^
Pre-pregnancy BMI (kg/m^2^)	20.4 ± 2.5	23.6 ± 3.3^*^	23.2 ± 3.9^*^
Pre-delivery BMI (kg/m^2^)	26.1 ± 2.7	29.3 ± 3.2^*^	28.8 ± 4.0^*^
Fetal growth restriction	0.62% (*N* = 36)	7.6% (*N* = 5)	21.8% (*N* = 12)^*^^#^
Placental abruption	0.052% (*N* = 3)	0.0% (*N* = 0)	7.3% (*N* = 4)^*^^#^
**Mode of delivery**			
Vaginal delivery	68.0% (*N* = 3,936)	7.6% (*N* = 5)^*^	7.3% (*N* = 4)^*^
Cesarean delivery	32.0% (*N* = 1,974)	92.4% (*N* = 61)^*^	92.7% (*N* = 51)^*^
Gestational age at delivery (weeks)	39.3 ± 1.1	37.3 ± 1.7^*^	32.1 ± 2.3^*^^#^
**Therapy**			
Combination of antihypertensive drugs		53.0% (*N* = 35)	78.2% (*N* = 43)^#^
Intravenous antihypertensive drugs		30.3% (*N* = 20)	50.9% (*N* = 28)%
**Newborn (*****N*** **=** **5,905)**			
Neonatal birth weight (g)	3,360.8 ± 387.3	2,975.3 ± 593.8^*^	1,870.6 ± 638.7^*^^#^
1-min Apgar	9.9 ± 0.6	9.8 ± 0.5^*^	8.9 ± 1.8^*^^#^
5-min Apgar	9.9 ± 0.3	9.9 ± 0.1	9.7 ± 0.8^*^^#^
NICU occupancy	1.36% (*N* = 79)	65.2% (*N* = 43)^*^	88.0% (*N* = 44)^*^^#^

*Statistically significant difference from normal pregnancy*

* *(P < 0.05)*.

*Statistically significant difference from late-onset PE*

# *(P < 0.05). NICU, neonatal intensive care unit; BMI, body mass index; PE, pre-eclampsia*.

Compared with healthy pregnant women, patients with late-onset and early-onset PE had the characteristics of older maternal age, multiple pregnancies and births with significant differences, and early-onset PE was more typical. Pre-pregnancy body mass index (BMI) in the PE groups was higher compared with the healthy group, and more marked weight gain during pregnancy made pre-delivery BMI much higher (*P* < 0.05). The incidences of FGR and placental abruption were higher in the PE groups compared with the healthy group, and related adverse pregnancy outcomes among patients with early-onset PE were significantly more common. Most patients with PE delivered via cesarean section. For patients with early-onset PE, it was often a requirement to reduce blood pressure to target levels using several antihypertensive drugs. The proportion of patients using intravenous antihypertensive drugs was also higher in the PE groups. Newborns in the early-onset PE group had a lower average birth weight and Apgar score because of earlier termination of pregnancy with increased admission to the neonatal intensive care unit (NICU). Fetuses in the late-onset group were delivered near or after term, but birth consequences of newborns were usually not as good as the healthy group.

### Association Between Maternal Lipid Profile, PE Onset, and Gestational Age at Delivery

Pregnant women with PE had more obvious lipid profile abnormalities in the second trimester compared with healthy pregnant women. TC, TG, and LDL-c levels in the early-onset PE group were significantly higher compared with the other two groups, while HDL-c was significantly reduced compared with the healthy group (Table 2). A correlation analysis showed that the levels of TC, TG, and LDL-c were negatively related to PE onset time and gestational week at delivery in the PE group (Table 3).

**Table 2:** Maternal lipid profiles in healthy pregnancy, late-onset PE, and early-onset PE.

		**Healthy (*N* = 5,789)**	**Late-Onset PE (*N* = 64)**	**Early-Onset PE (*N* = 55)**	***P***
TC	Median	6.15	5.56^*^	6.25^#^	0.000^*^
	(Quartiles 25, 75)	(5.51, 6.83)	(4.89, 6.21)	(5.10, 7.50)	
TG	Median	2.06	2.61^*^	3.12^*^	0.000^*^
	(Quartiles 25, 75)	(1.63, 2.60)	(1.92, 3.43)	(2.19, 4.23)	
HDL-c	Median	2.31	1.68^*^	1.70^*^	0.000^*^
	(Quartiles 25, 75)	(2.03, 2.76)	(1.51, 1.88)	(1.48, 2.01)	
LDL-c	Median	3.36	3.28	3.75^*^^#^	0.023^*^
	(Quartiles 25, 75)	(2.84, 3.91)	(2.79, 3.83)	(2.83, 4.64)	

*Results are presented as median ± interquartile range for continuous variables. Statistically significant difference from healthy pregnancy*

* *(P < 0.05)*.

*Statistically significant difference from late-onset PE*

# *(P < 0.05)*.

*TC, total cholesterol; TG, triglycerides; HDL-c, high-density lipoprotein cholesterol; LDL-c, low-density lipoprotein cholesterol; PE, pre-eclampsia*.

**Table 3:** Correlation between lipid profile and PE onset or gestational age at delivery.

	**PE onset**		**Gestational age at delivery**	
	**Spearman's correlation coefficient**	***P***	**Spearman's correlation coefficient**	***P***
TC	−0.326	0.000^*^	−0.336	0.000^*^
TG	−0.344	0.000^*^	−0.356	0.000^*^
HDL-c	0.113	0.179	0.119	0.154
LDL-c	−0.300	0.000^*^	−0.308	0.000^*^

* *P < 0.05. TC, total cholesterol; TG, triglycerides; HDL-c, high-density lipoprotein cholesterol; LDL-c, low-density lipoprotein cholesterol; PE, pre-eclampsia*.

### Risk Factors and Validation of the Early-Onset PE Model

**Figure 2:**
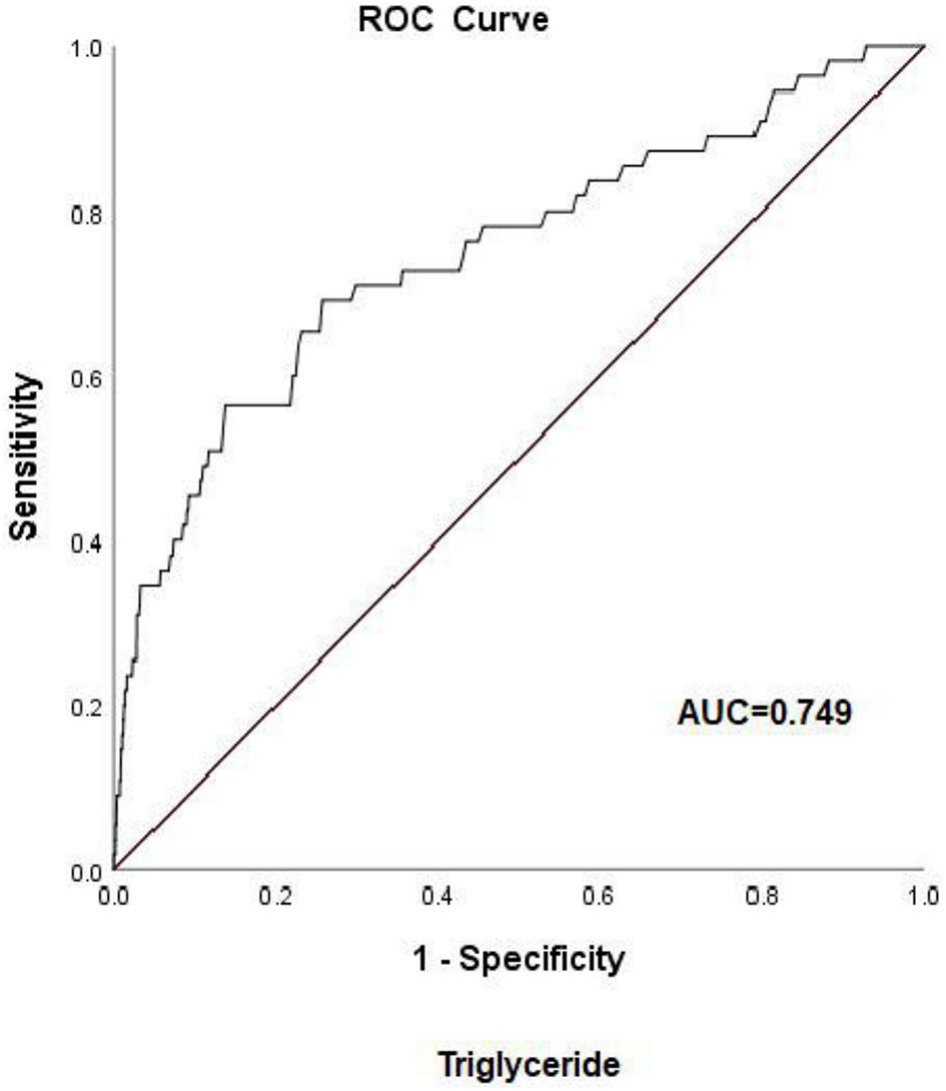
Receiver operating characteristic (ROC) curve analysis for predicting early-onset pre-eclampsia on the basis of maternal triglycerides (24–28 weeks of gestation). AUC, area under the curve.

**Figure 3:**
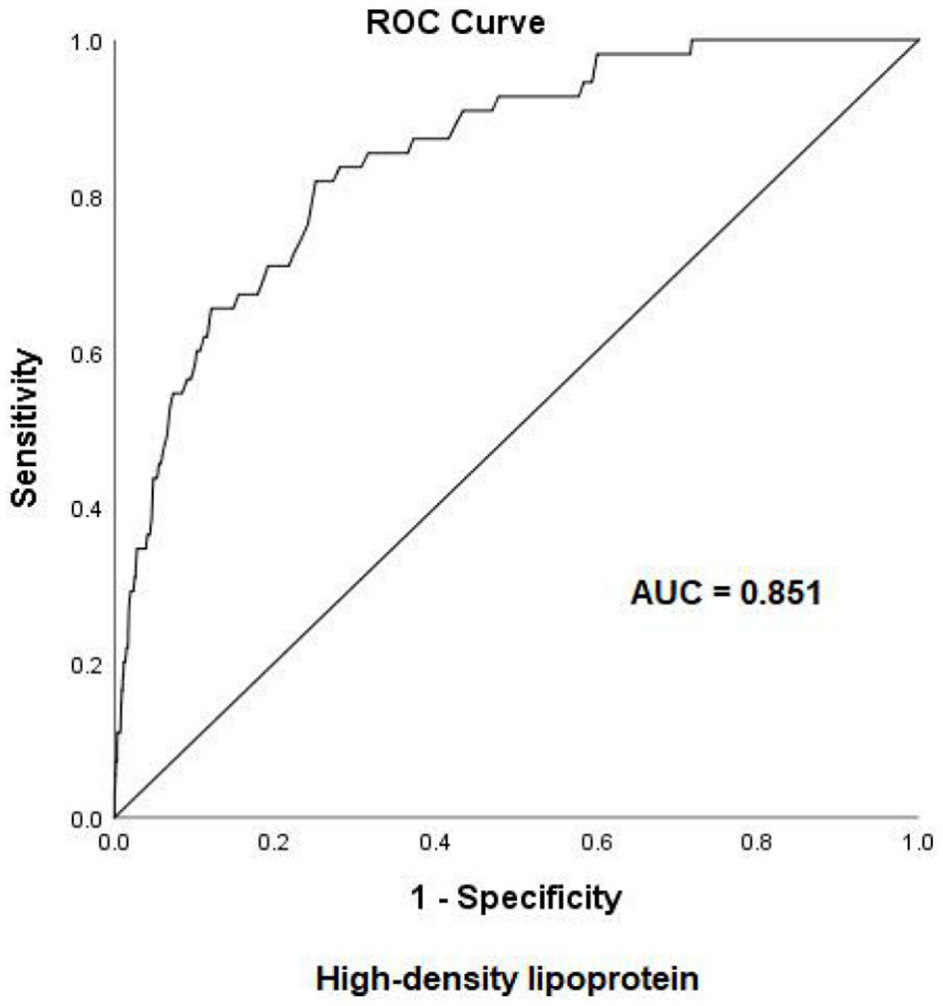
Receiver operating characteristic (ROC) curve analysis for predicting early-onset pre-eclampsia on the basis of maternal high-density lipoprotein (24–28 weeks of gestation). AUC, area under the curve.

Early-onset PE needs more attention due to adverse pregnancy outcomes. To examine the risk factors for early-onset PE, ROC curves were first introduced to define the cutoff value of lipid profiles, the area under the curve (AUC) was 0.749 for TG, and the cutoff value was 2.59 mmol/L with a specificity of 74.3% and a sensitivity of 69% (Figure 2). The AUC was 0.851 for HDL-c, and the cutoff value was 2.03 mmol/L with a specificity of 75% and a sensitivity of 82% (Figure 3), AUC for TC and LDL-c were both < 0.5 which were considered insufficient diagnostic potency (data not shown). HDL-c demonstrated a counter-trend, indicating that the smaller the value, the higher the probability of developing early-onset PE. The final regression model included five statistically significant predictors of early-onset PE: maternal age, parity, pre-pregnancy BMI, TG concentration, and HDL-c concentration (Table 4). A maternal age of ≥35 years had a greater risk [OR = 3.167 (χ^2^ =12.533, *P* < 0.05)]. Multipara seems to experience a greater risk of early onset PE [OR = 3.556 (χ^2^ = 14.813, *P* < 0.05)]. Pregnant women with a BMI of ≥25 and a second-trimester TG concentration of ≥2.59 mmol/L had an increased risk of developing early-onset PE [OR = 4.056 (χ^2^ = 18.414, *P* < 0.05) and OR = 2.059 (χ^2^ = 4.721, *P* < 0.05), respectively]. A second-trimester HDL-c concentration of ≤ 2.03 mmol/L indicated a higher risk of early-onset PE [OR = 7.371 (χ^2^ = 30.084, *P* < 0.05)].

**Table 4:** Multiple logistic regression of early-onset PE risk factors.

**Risk factors**		**β**	**SE**	**Wald **χ^2^****	***P***	**OR(95%CI)**
Age(years)	≥35	1.153	0.326	12.533	0.000^*^	3.167 (1.673–5.996)
	<35	0a				
Parity	≥1	1.269	0.330	14.813	0.000^*^	3.556 (1.864–6.785)
	0	0a				
Pre-pregnancy BMI(kg/m^2^)	≥25	1.400	0.326	18.414	0.000^*^	4.056 (2.140–7.687)
	<25	0a				
TG (mmol/L)	≥2.59	0.722	0.332	4.721	0.030^*^	2.059 (1.073–3.949)
	<2.59	0a				
HDL (mmol/L)	≤ 2.03	1.990	0.363	30.084	0.000^*^	7.314(3.593–14.893)
	**>**2.03	0a				

* *P < 0.05. PE, pre-eclampsia; OR, odds ratios; CI, confidence interval; BMI, body mass index; TG, triglycerides; HDL-c, high-density lipoprotein cholesterol; every 0a is defined as the calibration standard*.

**Figure 4:**
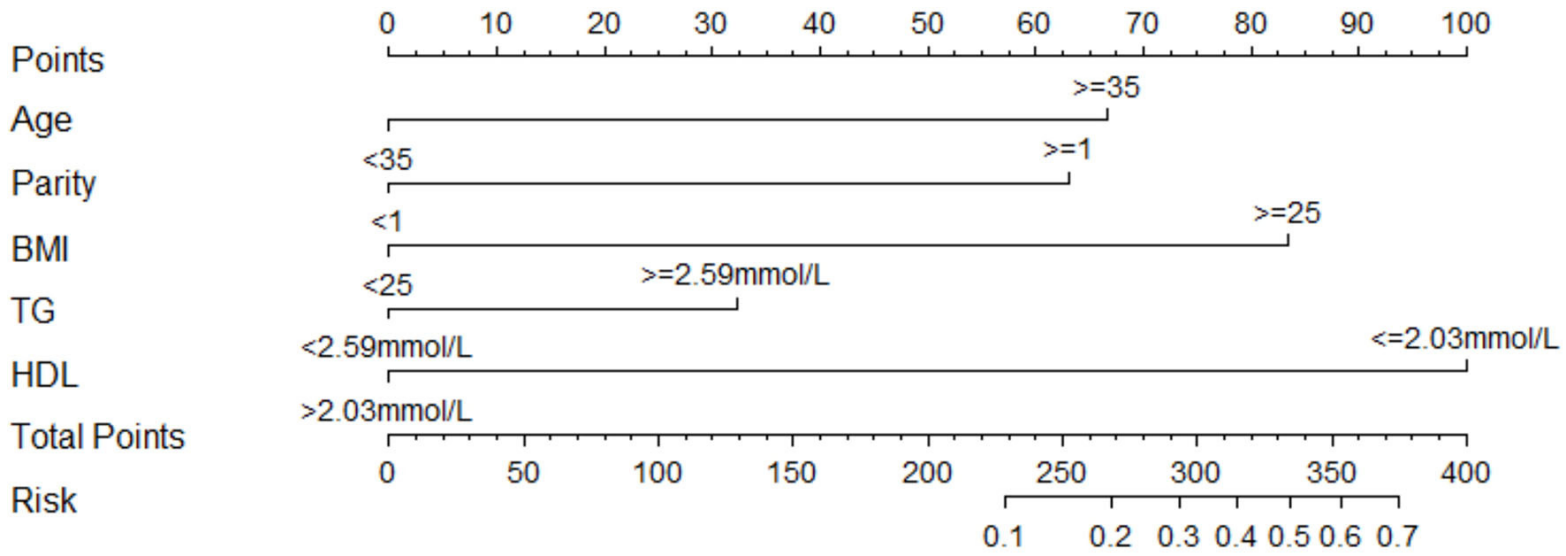
Nomogram to predict the probability of early-onset PE. Instructions to use the nomogram are as follows: (1) to obtain the nomogram-predicted probability, locate patient values on each axis; (2) draw a vertical line to the point axis to determine how many points are attributed for each variable value; (3) sum the points for all variables; (4) locate the sum on the total point line to assess the early-onset PE probability at the lower line of the nomogram.

**Figure 5:**
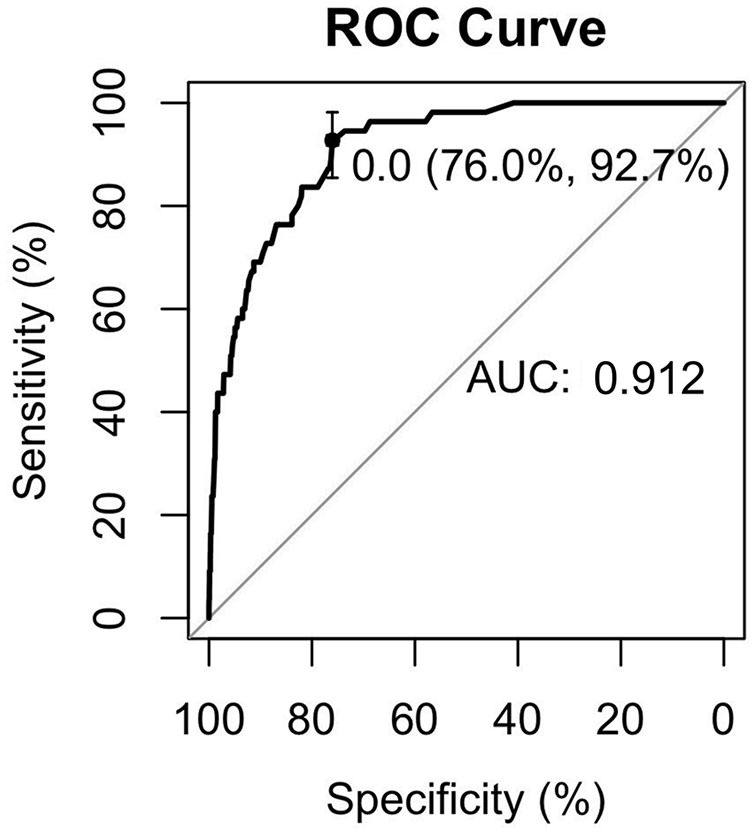
The receiver operating characteristic (ROC) curve was measured by bootstrapping for 1,000 repetitions. AUC, area under the curve.

**Figure 6:**
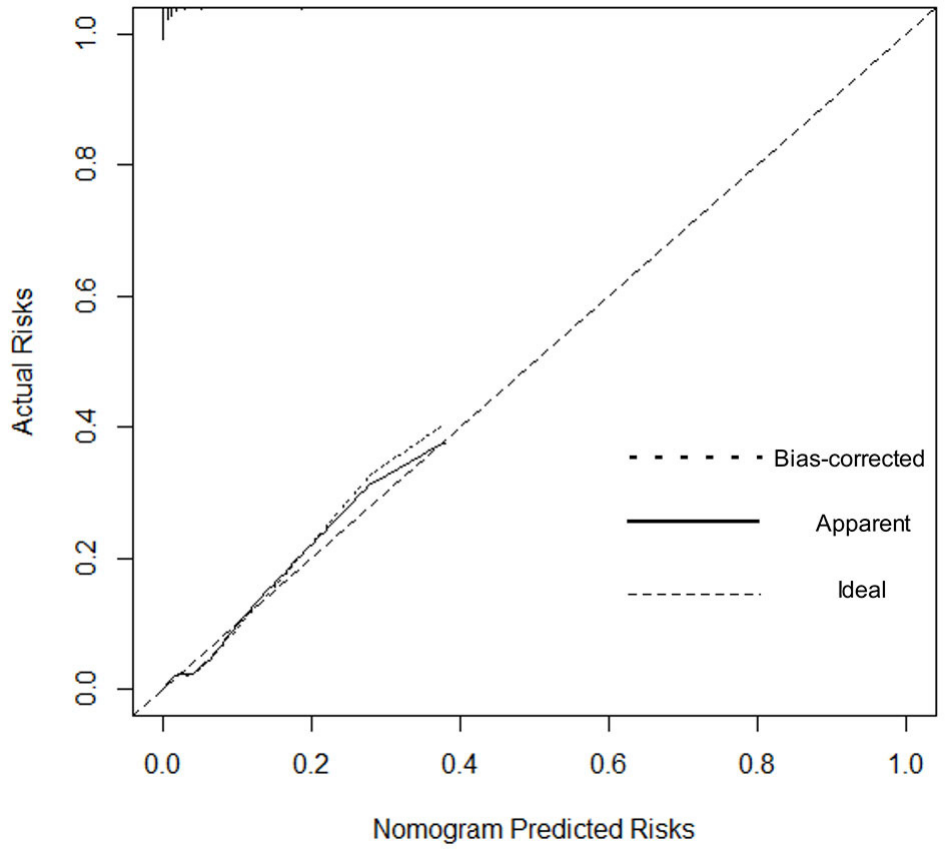
Calibration curve. The dashed line represents ideal predictions. The plot illustrates the accuracy of the best-fit model (Apparent) and the bootstrap model (Bias-corrected) for predicting early-onset pre-eclampsia.

**Figure 7:**
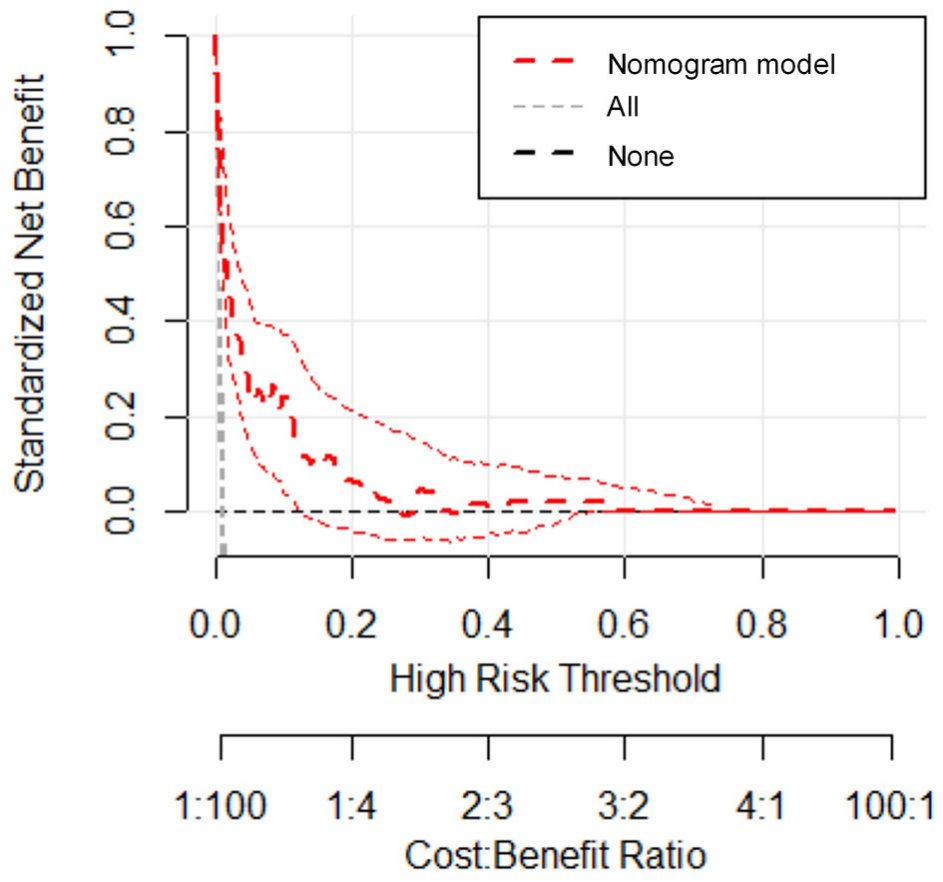
Decision curve analysis for the prediction model. The red line represents the prediction model. The gray dashed line assumes all patients have early-onset PE. The horizontal line assumes no patients have early-onset PE. The red dotted line represents the expected net benefit per patient relative to the nomogram prediction of early-onset pre-eclampsia with 95% confidence interval.

We estimated the probability of early-onset PE using the nomogram, which was developed using forward stepwise multiple logistic regression (Figure 4). The ROC curve analysis measured the performance of this nomogram, and the AUC of this model was 0.912 (Figure 5), indicating excellent diagnostic performance with a sensitivity of 92.7% and a specificity of 76% at the optimal cutoff value. The nomogram was further validated using internal bootstrap validation. A calibration curve demonstrated that our model showed good fitting and calibration with the ideal curve (Figure 6). Our decision curve analysis demonstrated good positive net benefit in the predictive model. which indicated a favorable clinical effect of the predictive model (Figure 7).

## Discussion

The pathogenesis of PE has not been completely elucidated. The entire pathobiological mechanism may have roots in lipid disorders, which may, in turn, be associated with obesity (22) or other conditions that can pathologically elevate blood lipids in pregnant women. Recently some scholars have proposed that PE is primarily a consequence of cardiovascular disorder, abnormal pre-pregnancy maternal cardiovascular function may lead to trophoblast malperfusion and may be the origin of PE (4). In uncomplicated pregnancies, there is a physiological progressive increase in the concentrations of lipids in maternal serum, which ensures proper fetal development (23); however, the mechanism by which pregnancy induces hyperlipidemia remains unclear. In our study, we observed and illustrated the abnormal second-trimester lipid profiling in PE patients, and the subsequent risk of early-onset PE. The excepted predictive value of those risk factors in the disease onset will be useful supplementary tool for clinical judgment, therefore, we constructed the nomogram to make it happen.

Excessive elevation in maternal TG concentration may have a role in the pathogenesis of PE; thus, this marker may identify pregnant women who are at risk of PE. Meanwhile, the significant increase in TG concentrations during gestation can be used as a biomarker of a lipoprotein atherogenic phenotype to identify women at risk of developing cardiovascular disease later in life (24). Researchers continue to investigate the mechanisms underlying the relationship between an abnormal lipid profile and PE onset. There is a 2–3-fold increase in serum TG concentration, which accumulates in the uterine spiral arteries and contributes to endothelial activation and damage (25). TG accumulation in endothelial cells can trigger a decrease in the release of prostaglandins and nitric oxide, leading to endothelial dysfunction (26). Oxidized cholesterol was also statistically significant among the markers of PE in an independent study using a global serum lipidomic approach to predict PE development later in pregnancy (27). This evidence indicates that an abnormal lipid profile would contribute to endothelial dysfunction in PE. It is also noteworthy that lipids can alter their function in response to reactive oxygen species (ROS) (28, 29). PE is associated with augmentation of oxidative stress and a reduction in endogenous antioxidant capacity, particularly that modulated by paraoxonase 1 (30). Apolipoproteins in HDL, mainly apolipoprotein A1, contribute to the enzymatic activity, stability, and function of paraoxonase 1 (31). Our study found that HDL-c levels in patients with PE were significantly lower compared with healthy patients, implying that HDL-c can be affected, leading to inactivation of paraoxonase 1. The consequences include loss of normal biological function, induction of the proinflammatory state, adhesion of macrophages to the vascular wall, an increase in atherosclerotic lesions, and other complications (32).

Early-onset PE is often accompanied by poor clinical outcomes, and treatment of early-onset PE is more difficult compared with late-onset PE. Techniques aim to prolong the duration of pregnancy and improve both maternal and neonatal outcomes. Our study shows that patients with early-onset PE have an older maternal age and multiple pregnancies and births. Pre-pregnancy BMI is also higher compared with the healthy group, and patients with early-onset PE gained more weight during pregnancy. Patients in the early-onset PE group were more likely to demonstrate FGR and had an increased risk of placental abruption. Cesarean section is often required to terminate pregnancy in patients with early-onset PE. The average gestational week of delivery was around 32 weeks, which is far from full term. Low birth weight among newborns accounted for entry of newborns to the NICU.

Nowadays, there is little research on the relationship between blood lipid levels especially the second-trimester lipids and early-onset PE. Some researchers believe that early-onset PE is associated with placental dysfunction, while late-onset PE develops predominantly due to metabolic disturbances, obesity, diabetes mellitus, lipid dysfunction, and inflammation. Garcia-Gonzalez et al. investigated and supported the hypothesis that maladaptation of maternal cardiovascular system precedes the development of PE (33). The new theory on the origin of PE demonstrated that cause of placental dysfunction in pre-eclampsia may be due to a suboptimal maternal cardiovascular performance resulting in uteroplacental hypoperfusion. Our results indicate that second-trimester lipid profiles of patients with PE reveal significantly increased serum levels of TG, TC, and LDL-c; these changes were more obvious in patients with early-onset PE. It is thought that HDL-c increases to protect the maternal vascular endothelium (34) and that this increase does not occur adequately in women with PE. We found that the HDL-c level in patients with PE in the second trimester was significantly lower compared with the healthy group; thus, this protective effect might be impaired in patients with PE. Our findings support the theory that PE share the same risk factors with cardiovascular disease, and abnormal lipid profiling is the signal of the pre-disease state.

To gain deeper insights into the changes involved in lipid metabolism and to highlight differences between abnormal lipid profiles and PE onset or gestational week of pregnancy termination, our results revealed that second-trimester changes in lipid profile are negatively correlated with gestational week; that is, the more obvious the increase, the earlier the onset time. The same negative correlation was observed with the time of termination in patients with PE. The results suggest that lowering abnormally elevated blood lipid levels is beneficial to the pathogenesis and treatment of PE. Because patients with PE are at a significantly increased risk of developing CHD in the later stages, we need to find a better strategy so that preventive and behavioral policies can be implemented. Interventional studies have been conducted and are ongoing for the prevention of PE or improvement of outcomes using lipid-modifying drug therapy (35). In other research, lipid apheresis has been explored as a possible therapeutic approach to prolong pre-eclamptic pregnancy (2, 36). However, due to limitations in the current clinical use of hypolipidemic drugs, these drugs have not been promoted. Early-onset PE deserves more clinical attention; thus, we investigated the risk factors related to its onset. We recognized that the risk of early-onset PE is significantly increased in elderly pregnant women, patients with multiple pregnancies, and patients with obesity. Some research indicated the prevalence of acquired cardiovascular disease during pregnancy is rising as older maternal age, obesity (37).Hence, our study showed convincing evidence for homology of PE and CHD.The maternal lipid profile suggests that when second-trimester TG concentration is ≥2.59 mmol/L or HDL-c is ≤ 2.03 mmol/L, the risk of early-onset PE is increased by 2.059 and 7.314-times, respectively. Since BMI and age may be the confounding factors to alter the lipid levels, we did the collinearity analysis to exclude the dependent relationships before introducing the final logistic regression analysis (data not shown), meanwhile, this results suggested that in early-onset PE patients without elder age and obesity still have abnormal blood lipid metabolism levels to increase the disease probability. To further verify the risk-factor model, the nomogram was introduced to calculate the overall probability of early-onset PE with an excellent diagnostic performance (AUC = 0.912, sensitivity = 92.7%, and specificity = 76%) and was validated internally using the bootstrap sampling method.

This prediction model is important for risk estimation and improving the clinical decision-making strategy for early-onset PE patients, which might serve as an essential early warning sign of severe PE onset. Pregnant women with a high score will be associated with a higher probability so that appropriate measures, including behavior, diet or drug management, can be taken.

Our research has clinically confirmed the adverse effects of abnormal second-trimester dyslipidemia in patients with PE, especially patients with early-onset PE, but further study of the mechanisms should be conducted to clarify how abnormal blood lipids affect PE pathogenesis. How best to identify high-risk populations and whether hypolipidemic drugs can significantly improve adverse pregnancy outcomes in patients with early-onset PE still requires clarification in high-quality randomized controlled trails. The results of these studies could make management of blood lipids an important measure to prevent and treat early-onset PE in the near future.

## Limitations

The main limitation of the present study was the comparatively small sample size of the groups and the single observational study design. As a major obstetric disease center in Zhejiang Province, many patients with severe PE come from referrals and lack standardized supervision and management during pregnancy. Second, the nomogram lacked robust external validation. Therefore, multi-center research and data sharing construction and further validation are important means to solve the abovementioned limitations.

## Data Availability Statement

The raw data supporting the conclusions of this article will be made available by the authors, without undue reservation.

## Ethics Statement

The studies involving human participants were reviewed and approved by Ethics Committee of Women's Hospital, Zhejiang University School of Medicine. The patients/participants provided their written informed consent to participate in this study.

## Author Contributions

JLi was in charge of conceptualization, design of the study, data curation and analysis, and the drafting and revision of the manuscript. JLu contributed to design of the study, sample analysis, data acquisition, and interpretation. MW and WH were in charge of data acquisition, the methodology and interpretation. NJ and XL participated in design of the study and critical revision of the manuscript. BZ participated in supervision and data acquisition. QL was in charge of conceptualization, design of the study, data curation and analysis, manuscript review, and editing. All authors contributed to the article and approved the submitted version.

## Conflict of Interest

The authors declare that the research was conducted in the absence of any commercial or financial relationships that could be construed as a potential conflict of interest.

## Publisher's Note

All claims expressed in this article are solely those of the authors and do not necessarily represent those of their affiliated organizations, or those of the publisher, the editors and the reviewers. Any product that may be evaluated in this article, or claim that may be made by its manufacturer, is not guaranteed or endorsed by the publisher.

## Abbreviations

PE: Pre-eclampsia
TC: Total cholesterol
TG: Triglycerides
LDL-c: Low-density lipoprotein cholesterol
HDL-c: High-density lipoprotein cholesterol
ROC: Receiver operating characteristic curve
CHD: Coronary heart disease
FGR: Fetal growth restriction
OR: Odds ratio
CI: Confidence interval
NICU: Neonatal intensive care unit
AUC: Area under the curve.
